# Membrane Transporters in Human Parotid Gland-Targeted Proteomics Approach

**DOI:** 10.3390/ijms20194825

**Published:** 2019-09-28

**Authors:** Joanna Lapczuk-Romanska, Diana Busch, Ewa Gieruszczak, Agnieszka Drozdzik, Katarzyna Piotrowska, Robert Kowalczyk, Stefan Oswald, Marek Drozdzik

**Affiliations:** 1 Department of Experimental and Clinical Pharmacology, Pomeranian Medical University, ul. Powstancow Wlkp 72, 70-111, Szczecin, Polande.gieruszczak@gmail.com (E.G.); 2 Department of Clinical Pharmacology, University Medicine of Greifswald, Felix-Hausdorff-Str. 3, 17489 Greifswald, Germany; diana.busch@uni-greifswald.de (D.B.); stoswald@uni-greifswald.de (S.O.); 3 Department of Integrated Dentistry, Pomeranian Medical University, ul. Powstancow Wlkp 72, 70-111 Szczecin, Poland; agdro@pum.edu.pl; 4 Department of Physiology, Pomeranian Medical University, ul. Powstancow Wlkp 72, 70-111 Szczecin, Poland; 5 Department of Maxillofacial Surgery, Pomeranian Medical University, ul. Unii Lubelskiej 1, 71-252 Szczecin, Poland; robertkowalczyk@pum.edu.pl

**Keywords:** protein abundance, gene expression, membrane transporters, human, parotid gland, saliva

## Abstract

Salivary glands provide secretory functions, including secretion of xenobiotics and among them drugs. However, there is no published information about protein abundance of drug transporters measured using reliable protein quantification methods. Therefore, mRNA expression and absolute protein content of clinically relevant ABC (*n* = 6) and SLC (*n* = 15) family member transporters in the human parotid gland, using the qRT-PCR and liquid chromatography‒tandem mass spectrometry (LC−MS/MS) method, were studied. The abundance of nearly all measured proteins ranged between 0.04 and 0.45 pmol/mg (OCT3 > MRP1 > PEPT2 > MRP4 > MATE1 > BCRP). mRNAs of *ABCB1*, *ABCC2*, *ABCC3*, *SLC10A1*, *SLC10A2*, *SLC22A1*, *SLC22A5*, *SLC22A6*, *SLC22A7*, *SLC22A8*, *SLCO1A2*, *SLCO1B1*, *SLCO1B3* and *SLCO2B1* were not detected. The present study provides, for the first time, information about the protein abundance of membrane transporters in the human parotid gland, which could further be used to define salivary bidirectional transport (absorption and secretion) mechanisms of endogenous compounds and xenobiotics.

## 1. Introduction

Membrane transporters facilitate the movement of endogenous molecules as well as drugs and other xenobiotics across cell membranes and therefore are critical determinants of cellular homeostasis and drug/xenobiotics handling. They can be broadly categorized into two superfamilies: the solute carrier (SLC) transporters comprising over 400 integral membrane proteins subdivided into 50 families and the ATP-binding cassette (ABC) superfamily consisting of about 50 members subdivided into 7 families (from ABCA to ABCG). SLC transporters facilitate mostly the cellular uptake of many endogenous compounds and drugs (antibiotics, antivirals, chemotherapeutics). On the contrary, ABC transporters facilitate mainly the efflux of cationic, hydrophobic and amphoteric substances, including drugs (immunosuppressants, chemotherapeutics, macrolides) [1]. 

Membrane proteins participating in drug transport are widely expressed in pharmacokinetically important tissues such as liver, intestine, kidney and blood-tissue barriers [2]. However, there are only a few reports showing that some members of the SLC and ABC families are also expressed in salivary glands, where they may be associated with absorption (SLC) and secretion (ABC) of drugs and endogenous molecules in this organ [3,4]. In most cases, only data about ion and water transporters but not on relevant drug transporters were provided. The available information is mostly based on mRNA expression levels and protein abundance estimated through immunological methods (mainly Western blot and immunohistochemistry). However, these analytical techniques have some limitations, i.e., a high number of PCR cycles may generate expression signals that can easily cause misleading observations, and application of polyclonal, non-specific antibodies, can produce false positive results.

Within the members of the ABC family, P-glycoprotein (MDR1, ABCB1), MRP1 (ABCC1) and MRP2 (ABCC2) were detected in human salivary glands at mRNA level (*ABCB1*, *ABCC1*-*ABCC12 ABCG2*) [5,6] and, using antibody-based methods (P-glycoprotein, ABCC1, ABCC2), also on protein level. P-glycoprotein, MRP1 and MRP2 were identified at basolateral and luminal membrane of duct cells, indicating their impact on salivary gland functions and drug accumulation or/and excretion [7,8,9]. Similarly, some members of the SLC family were detected in salivary glands. mRNA expression studies provided information on the expression of the following SLC transporters: *SLC15A2*, *SLCO1A2*, *SLC22A1*, *SLC22A3*, *SLC22A4*, *SLC22A5*, *SLC22A6*, *SLC22A7* and *SLC22A18* [5] and *SLCO1A2* [6]. Immunohistochemical staining showed OAT1-4 expression in whole salivary glands [10] and OCT3 on apical and basolateral membranes of salivary gland acinar cells [11]. 

The published studies indicate that human salivary glands are endowed with membrane transporters, from both ABC and SLC families, which provide efflux and influx functions, regulating membrane shift of their substrates. However, the above studies report mainly mRNA expression data, also at a very low level (which is not always translated into significant levels of the respective proteins), and a limited number of protein levels (using antibody-based methods with all limitations of those assays). Therefore, the aim of this study was to evaluate protein abundance of 21 membrane transporters (6 ABC and 15 SLC family members) in the human parotid gland using highly sensitive high-quality liquid chromatography‒tandem mass spectrometry (LC−MS/MS) method. To the best of our knowledge, this is the first study which has comprehensively covered expression information (mRNA and protein) of clinically relevant drug transporters in human salivary glands. The results of the present study complement information about transporter functions in human salivary glands and may also contribute to a better understanding of the role of salivary glands in oral health and saliva diagnostics [12]. 

## 2. Results

**Figure 1:**
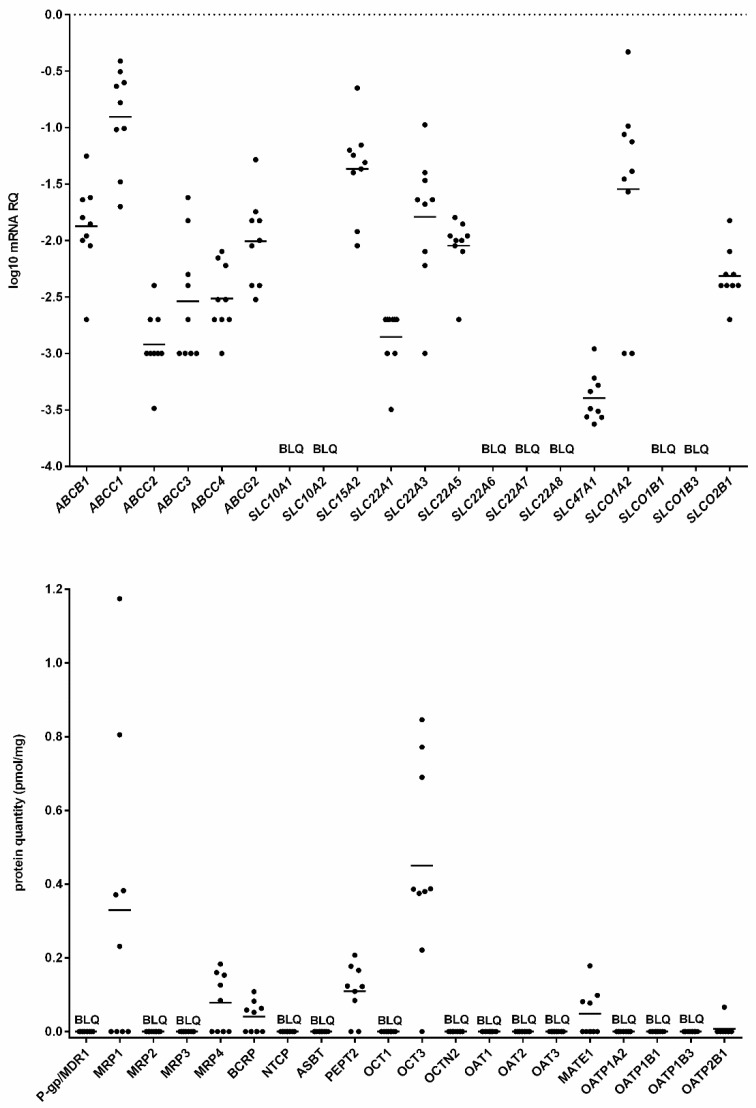
A scatter plot of the measured gene expression (**top**) and protein abundance (**bottom**) of ATP-binding cassette (ABC) and solute carrier (SLC) transporters in human parotid glands. Lines indicate population means of the sets of data. mRNA level (log-transformed values) of the analyzed genes was expressed as relative amounts to the mean of five housekeeping genes (*ACTB, GAPDH, PPIA, RPLP0, RPS9*). BLQ—below quantification limit.

All ABC family transporters genes included into this analysis were expressed at detectable levels (C_T_ < 35) in salivary glands, with the highest level of *ABCC1.* In parallel to mRNA expression, MRP1 (ABCC1) was the most abundant protein, followed by MRP4 (ABCC4) and BCRP (ABCG2). MDR1 (ABCB1), MRP2 (ABCC2) and MRP3 (ABCC3) could not be detected in any of the studied samples, as they were below the lower limit of quantification (<0.04 pmol/mg) (Figure 1, Table S1, Table S2). 

Among SLC transporters, only *SLCO1A2*> *SLC15A2*> *SLC22A3*> *SLC22A5*> *SLCO2B1*> *SLC22A1* were expressed (mRNA levels above the limit of quantification) in parotid glands, whereas OCT3, PEPT2, MATE1 and OATP2B1 (in one out of nine analyzed samples) were the only transporters detected at protein levels (Figure 1, Table S1, Table S2). Na^+^/K^+^-ATPase, a reference membrane protein and marker of basolateral membrane, was detected in all the analyzed tissue samples (*n* = 9) at both mRNA and protein levels (Table S1, Table S2). 

**Figure 2:**
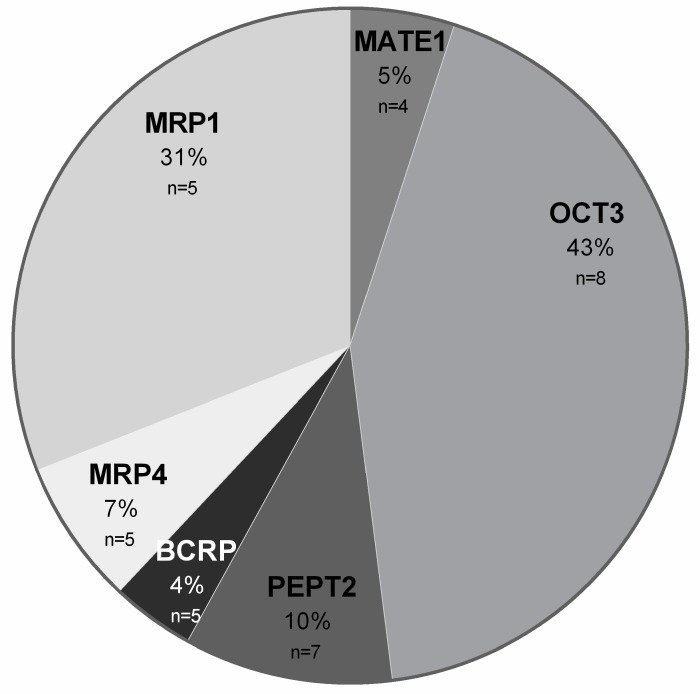
Pie chart/percentage of ABC and SLC transporters in salivary glands. Mean percentage contribution of analyzed transporters in salivary glands.

Based on the protein data, the abundance of ABC and SLC transporters in salivary glands was as follows: OCT3 > MRP1 > PEPT2 > MRP4 > MATE1 > BCRP (Figure 2). 

A significant negative correlation between mRNA level and protein abundance in salivary gland was shown for BCRP. The remaining transporters did not demonstrate significant mRNA expression/protein abundance correlations (Table 1). Analyzing the correlations of protein abundance among the studied transporters, significant negative correlations between MRP1/MRP4 and MRP4/Na^+^/K^+^-ATPase could be determined (Supplementary Table S3).

**Figure 3:**
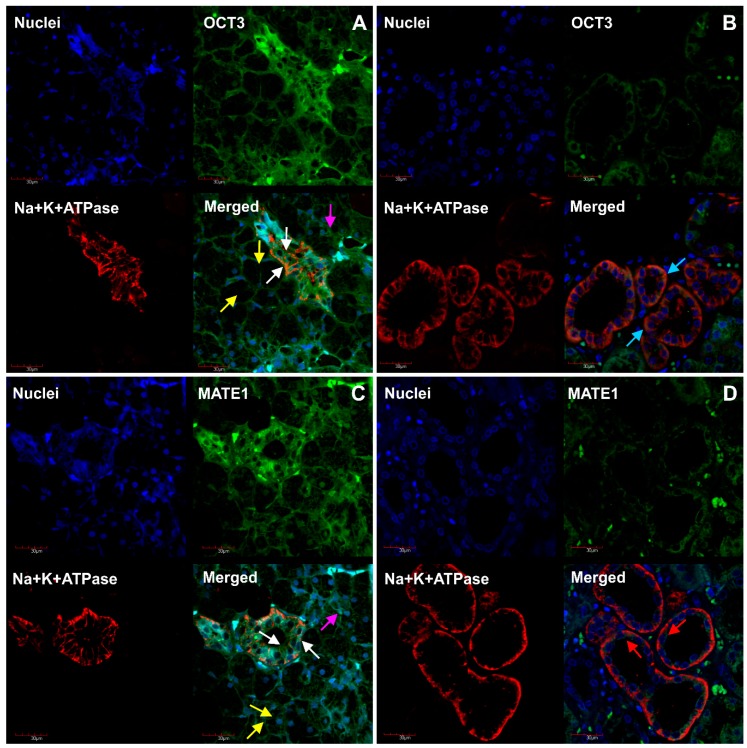
Immunofluorescence staining of OCT3 and MATE1 in salivary glands and kidney cortex. (**A**) OCT3 (green panel) expression at the basolateral and apical membrane of serous and mucous acinar cells and in duct cells. (**B**) OCT3 (green panel) expression at basolateral membrane of kidney proximal tubule. (**C**) MATE1 (green panel) detection at the basolateral and apical membrane of serous and mucous acinar cells and in duct cells. (**D**) MATE1 (green panel) detection at apical membrane of kidney proximal tubule. Na^+^/K^+^-ATPase (red panel **A**–**D**) was used as the basolateral membrane marker. Nuclei are stained blue (**A**–**D**). Yellow arrow—apical and basolateral membrane of mucous acinar cells; white arrow—apical and basolateral membrane of duct cells; pink arrow—serous acinar cells; blue and red arrow—basolateral and apical membrane of proximal tubule cells, respectively.

**Figure 4:**
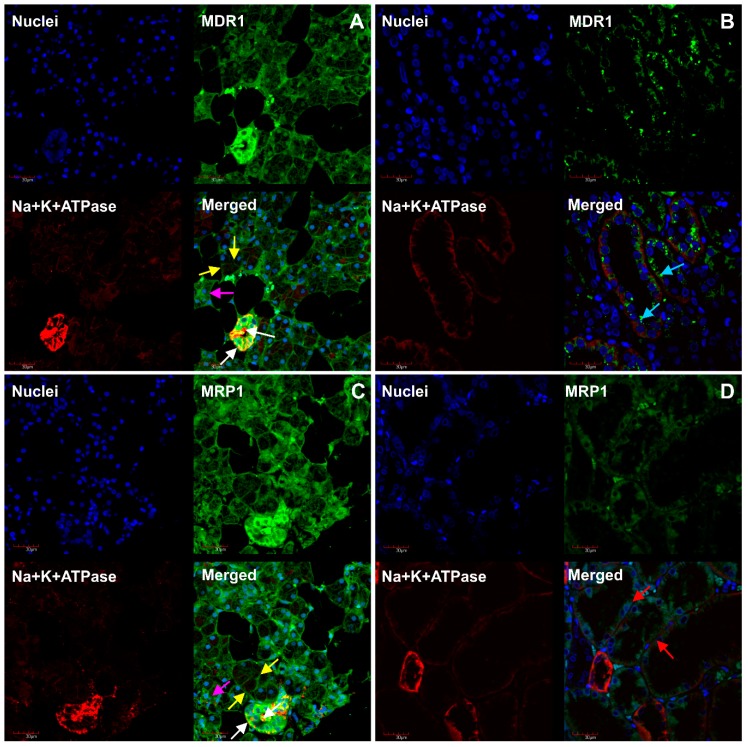
Immunofluorescence staining of MDR1/P-gp and MRP1 in salivary glands and kidney cortex. (**A**) MDR1 (green panel) expression at the basolateral and apical membrane of serous and mucous acinar cells and in duct cells. (**B**) MDR1 (green panel) expression at apical membrane of kidney proximal tubule. (**C**) MRP1 (green panel) detection at the basolateral and apical membrane of serous and mucous acinar cells and in duct cells. (**D**) MRP1 (green panel) detection at basolateral membrane of kidney distal tubule. Na^+^/K^+^-ATPase (red panel **A**–**D**) was used as the basolateral membrane marker. Nuclei are stained blue (**A**–**D**). Yellow arrow—apical and basolateral membrane of mucous acinar cells; white arrow—apical and basolateral membrane of duct cells; pink arrow—serous acinar cells; blue and red arrow—apical membrane of proximal tubule cells and basolateral membrane of distal tubule cells, respectively.

Immunofluorescence staining showed OCT3 and MATE1 expression at the basolateral and apical membrane of serous and mucous acinar cells and in duct cells; however, immunofluorescence staining of MATE1 was weaker in comparison to OCT3 (Figure 3). Similarly, P-gp and MRP1 proteins were expressed at both apical and basolateral membrane of serous and mucous acinar cells and in duct cells (Figure 4). Na^+^/K^+^-ATPase was used as the basolateral membrane marker in acinar cells and confirmed the expected localization.

## 3. Discussion

To the best of our knowledge, this is the first study providing information about protein abundance of drug transporters in salivary glands using the targeted proteomic method (LC–MS/MS). The study also verifies and complements the previously reported information about the mRNA and protein level of these transporters measured using semiquantitative methods. Our study confirms protein abundance of ABCC1, ABCC4 and ABCG2 (previously observed at mRNA and protein level measured using Western blot and immunohistochemistry methods). However, we were not able to demonstrate the presence of P-glycoprotein (MDR-1), MRP2 and MRP3 (although seen in all cases at mRNA level). Therefore, the broad expression of ABC family transporter in salivary glands reported by Nishimura and Naito [5] could not be confirmed. Our finding confirms the current notion that mRNA-based studies may overestimate the biological role of studied genes, due to methodological aspects or engagement of posttranscriptional factors. Low expression of target genes is usually reported when using a high number of PCR cycles, as they may produce expression signals that can easily cause misleading observations. For example, initial reports showed intestinal expression of the liver-specific uptake transporters *SLCO1B1* and *SLCO1B3* [13]. Lack of correlation between mRNA and protein levels could also explain the aforementioned discrepancies. For example, in the gastrointestinal tract, significant mRNA/protein correlations were not determined for *ABCB1*/MDR1 (P-glycoprotein), *ABCC2*/MRP2 and *ABCC3*/MRP3; however, a positive correlation was found for *ABCG2*/BCRP [14]. As revealed for gastrointestinal tract, miRNA could be involved in posttranscriptional regulations, e.g., as shown for intestinal *ABCC3*/MRP3, where miR-192-5p was demonstrated to downregulate protein abundance of MRP3 [15]. The present study, using parotid gland tissue, did not demonstrate any significant positive correlations for the studied ABC family transporters; a negative correlation was observed for *ABCG2*/BCRP. However, because the number of samples available for correlation analysis was limited, these observations should be considered as preliminary.

The ABC transporters (P-glycoprotein, MRP1, MRP2) were also identified in salivary glands using antibody-based techniques [7,8,9]. Our targeted proteomic study confirmed protein abundance of MRP1. P-glycoprotein and MRP2 were not detected. Among all analyzed drug transporters, MRP1 was the most abundant protein in salivary glands. The present study also confirmed the expression of MRP1 using the immunofluorescence method, with its colocalization on basolateral and apical membranes of the salivary gland cells. This finding suggests the role of MRP1 in preventing drug accumulation within salivary glands and in drug secretion into saliva. Whether the substrates of MRP1 (e.g., anticancer drugs—doxorubicin, methotrexate, cyclophosphamide or HIV protease inhibitors—efavirenz, nevirapine and antimicrobial agents—ciprofloxacin) [16] are excreted into saliva via this transporter protein has to be investigated. Nevertheless, MRP-1 salivary efflux function can be potentially involved in oral mucositis produced by methotrexate [17], doxorubicin [18], cyclophosphamide [19] (via mediating the excretion of the drugs from blood into saliva) or treatment of bacterial pathogens within the gland (ciprofloxacin) [20]. It should be stated that both doxorubicin and methotrexate are also substrates of BCRP [16], and this efflux transporter defined in our study in salivary glands could contribute to their biological effects produced in the oral cavity. Our study with monoclonal antibody directed against P-glycoprotein visualized the transporter in salivary glands, which is contrary to our proteomic study, but in line with immunohistochemical data of other authors [7,8,9]. P-glycoprotein was expressed both on the apical and basolateral membrane of salivary gland cells. Western blot and quantitative immunohistochemistry possess some limitations (compared with the targeted proteomics method), mainly related to uncertain specificity of the used antibody, i.e., cross-reactivity with other proteins or even a lack of functionality, as well as not too well defined linear analytical range or poor reproducibility [21]. The LC–MS/MS based proteomic method is more specific and partly more sensitive. This method allows simultaneous quantification of several ADME proteins [22,23,24,25] and can be characterized in an analytical manner, i.e., distinct data on accuracy and precision are available [26,27,28]. Thus, after certain method validation, targeted proteomic assays are justified to be considered as more reliable. In the case of P-glycoprotein determination, we analyzed simultaneously two different peptides, which are so far the best established P-glycoprotein peptides (AGAVAEEVLAAIR and IATEAIENFR). The two analyzed peptides showed no analytical signals; therefore, we concluded that the protein abundance is below the lower limit of quantification. To verify the findings produced using the two above methods, further in vitro functional studies with P-glycoprotein substrates in salivary cells can be considered. 

Similarly to ABC transporters, the existing information about mRNA content of SLC family transporters should be revised. The published expression data list the following SCL transporters in salivary glands: *SLC15A2*, *SLCO1A2*, *SLC22A1*, *SLC22A3*, *SLC22A4*, *SLC22A5*, *SLC22A6*, and *SLC22A7* [5,6], which is in keeping with the present study results. However, we did not measure *SLC22A4*, and contrarily, *SLC22A6* was below the quantification limit. Protein information based on immunohistochemistry reported the presence of OAT1-4 and OCT3 [5,6,11]. The present study did not confirm expression of any of the aforementioned OATs, and other proteins where mRNA expression was detected, i.e., OATP1A2 and OCT1, as well as OCTN2. The liver specific transporters *SLC10A1*/NTCP, *SLCO1B1*/OATP1B1 and *SLCO1B3*/OATP1B3, as well as intestinal *SLC10A2*/ASBT, were also not detected in the present study at mRNA or protein levels. 

We were also able to identify the protein abundance of the transporters, which have not been reported yet, namely PEPT2 and MATE1. Peptide transporter 2 (PEPT2) is highly expressed at the apical membrane of proximal tubules, where it can be involved in the reabsorption of di- and tripeptides and peptide-like drugs [29], e.g., cephalosporin, cloxacillin and aminopenicillin [30]. These antibiotics demonstrated high salivary gland and saliva accumulation [31,32]. Whether PEPT2 directly plays a role in salivary gland accumulation of these drugs has yet to be investigated.

The method used in this study was also applied to quantify transporter proteins in the human gastrointestinal tract and liver [14,33]. Protein abundance of most of the transporters defined in the small intestine ranged between 0.2 and 1.6 pmol/mg, with the exception of those of OCT3 (<0.1 pmol/mg). In the liver, the amount of the detected transporter proteins was substantially higher, mostly over 20 pmol/mg, with the exception of MRP1 and OCT3 (less than 3 pmol/mg). The protein level of the transporters defined in the human parotid gland was lower, and ranged between 0.04 and 0.45 pmol/mg. However, the observed protein abundance of OCT3 in the salivary gland seems to be higher than in the jejunum (0.45 ± 0.27 pmol/mg), but lower than in the liver (2.92 ± 3.56 pmol/mg, detected in four out of nine subjects in the liver vs. eight out of nine in the salivary gland).

The present study demonstrates large interpersonal variability in ABC and SLC transporter protein abundance in the human parotid glands, as was demonstrated in other tissues/organs, e.g., liver or intestine [14,34,35,36,37]. It should be noted that only reference transporter protein, i.e., Na^+^/K^+^-ATPase, was observed in all studied tissue samples (thus confirming effective isolation of the membrane fraction); all the other ABC and SLC transporters detected were not present at protein levels in some subjects. This finding could explain interpersonal variability in drug excretion into saliva.

Our study provides for the first time reliable quantitative information on drug transporters in the human salivary gland. In this study, liquid chromatography coupled to tandem mass spectrometry (LC–MS/MS) was implemented, which is widely considered the preferred method for the identification and quantification of proteins [38]. The targeted proteomics results from the present study, therefore, complement and enable the reanalysis of the existing information about the role of drug transporters in handling of drugs in salivary glands. However, a controversy regarding the expression/function of P-glycoprotein in salivary glands appears, i.e., the lack of P-glycoprotein in salivary glands measured using the LC–MS/MS method and the reported positive immunohistochemical and immunofluorescence signals. Therefore, reports on the biological role of the transporter, such as the contribution of *ABCB1* gene (coding for P-glycoprotein) polymorphism to the modulation of salivary secretion of digoxin [39] or paracetamol transport in salivary glands [40] should be analyzed with caution. Other verification methods, such as studies on P-glycoprotein mediated transport in salivary gland cell lines may contribute to the final definition of the role of this transporter protein in salivary glands.

The present study confirms the expression and abundance of OCT3 with its co-localization on both basolateral and apical membranes and thus confirms the results of Lee et al. [11] on OCT3 transporter involvement in metformin transport in salivary glands. Our results also provide prerequisites on the transporter pathway of metformin, as protein abundance of MATE1 and its colocalization on basolateral and apical membrane was defined in the salivary gland. Both OCT3 and MATE1 can provide the uptake and excretion mechanism (respectively) of this drug in salivary glands, similarly to OCT1, OCT2 and MATE1 and MATE2 in the liver and kidneys [41]. The localization of OCT3 and MATE1 on both apical and basolateral membranes suggests that transport in both directions is possible, depending on the functional state of the gland as well as the drug concentration gradient, which was also postulated by Lee et al. [11]. In the current study we used polyclonal antibodies for OCT3 and MATE1 localization. To confirm the specificity (and lack of cross-reactivity) of these antibodies, we used kidney tissue cortex as a positive control in which OCT3 is localized only on basolateral membrane and MATE1 on apical membrane of proximal tubule. MATE1 as well as OCT3 mediate the transport of procainamide and this function, as in the case of metformin, which could explain its transport into saliva and the side effects produced in the oral cavity, i.e., bitter taste [42].

Another compound that was reported to undergo potential OCT3-mediated accumulation in salivary glands is methamphetamine and its metabolites [43]. Whether the high salivary caffeine concentrations are also associated with transporter expression and function still remains uncertain and should be investigated in the future [44]. A potential physiological role of OCT3 was reported in patients suffering from Sjogren’s syndrome. That is to say, mRNA and protein expression of OCT2 and OCT3 were significantly diminished, which was speculated to be associated with disturbed maintenance of high histamine levels in these patients [45]. 

The results of the present study may also explain findings suggesting that phase II metabolites have not generally been detected in saliva [46]. It is known that phase II metabolites, resulting from glucuronidation and sulfonation, are substrates of MRP2 and MRP3, which were not detected in the salivary glands. However, it should be stated that some glucuronides can be transported by MRP1 and MRP4 (revealed in the study in salivary glands). MRP4 participates among others in the transport of methotrexate and cephalosporins [47] and thus produces similar effects to the abovementioned characteristics of MRP1 related to oral cavity mucositis and the salivary secretion of cephalosporins. 

The existing information about the salivary secretion of drugs suggests the potential application of saliva in therapeutic drug monitoring, mainly due to simple and noninvasive sampling. It is accepted that saliva reflects the free non-protein bound drug concentration. However, as evidenced in the present study, salivary expression of drug transporters may modulate the rate of salivary drug secretion (also variable due to altered activity of the drug transporters subjected to endo- and exogenous inhibitors/inducers). The engagement of the drug transporters in salivary glands may thus constitute one of the limitations (apart from, e.g., physico-chemical drug properties, blood pH and blood protein binding) for therapeutic drug monitoring [46,48,49]. 

A considerable limitation of our study was the small number of salivary gland specimens included in the investigation. It should also be stated that, due to limited sample size, we evaluated the transporter abundance in the membrane fraction, which enriched the concentration of the proteins in comparison to total protein lysate.

The results of the present study suggest that salivary glands are rather poorly endowed in uptake transporters, as only OCT3 and PEPT2 could be detected at protein levels, whereas protein abundance of several efflux transporters was found, i.e., MRP1, MRP4, BCRP and MATE1. These findings could be further used to understand the salivary secretion mechanisms of endogenous compounds, drugs and other xenobiotics.

## 4. Materials and Methods

### 4.1. Parotid Gland Specimens

Parotid salivary gland tissue specimens were obtained from otherwise healthy patients diagnosed with tumor mixtus (6 females and 3 males, aged 40–60). The healthy tissues (without pathological picture during histopathological examination) of the parotid salivary glands were obtained from dissected glands. Resected tissues were immediately collected in RNAlater solution (Applied Biosystems, Darmstadt, Germany) for RNA analysis or snap-frozen in liquid nitrogen for protein analysis. Samples were stored at 80 °C until further analysis/use. Adjacent tissue was also embedded in formalin for immunohistochemistry. The study was approved by the Local Bioethics Committee of Pomeranian Medical University (resolution No. KB-0080/22/09) from 23 February 2009; project identification code 127/01/18. 

### 4.2. mRNA Isolation and Quantitative Real Time PCR

Total RNA from approximately 30‒50 mg of parotid gland tissue was isolated using a Direct-zol^™^ RNA MiniPrep Kit (Zymo Research, Irvine, CA, USA). cDNA was performed from 500 ng of total RNA in 40 µL of reaction volume using a SuperScript VILO cDNA Synthesis Kit (Thermo Fisher, Waltham, MA, USA), according to the manufacturer’s instructions. The gene expression level of analyzed transporter genes was examined in duplicate using the ViiA7 Real-Time PCR System for: *ABCB1* (Hs00184500_m1), *ABCC1* (Hs01561502_m1), *ABCC2* (Hs00166123_m1), *ABCC3* (Hs00978473_m1), *ABCC4* (Hs00988717_m1), *ABCG2* (Hs01053790_m1), *SLC10A1* (Hs00161820_m1), *SLC10A2* (Hs01001557_m1), *SLC15A2* (Hs01113665_m1), *SLC22A1* (Hs00427552_m1), *SLC22A3* (Hs00222691_m1), *SLC22A5* (Hs00929869_m1), *SLC22A6* (Hs00537914_m1), *SLC22A7* (Hs00198527_m1), *SLC22A8* (Hs00188599_m1), *SLC47A1* (Hs00217320_m1), *SLCO1A2* (Hs00366488_m1), *SLCO1B1* (Hs00272374_m1), *SLCO1B3* (Hs00251986_m1), *SLCO2B1* (Hs01030353_m1). Relative gene expression was calculated using the 2^−ΔCt^ method. The Ct value of each target gene was determined and normalized to the mean value for five housekeeping genes: *PPIA* (Hs04194521_s1), *RPLP0* (Hs99999902_m1), *RPS9* (Hs02339424_g1), *ACTB* (Hs99999903_m1) and *GAPDH* (Hs02786624_g1). 

### 4.3. Protein Quantification Using LC–MS/MS

Sample preparation and protein quantification of MDR1 (P-gp, ABCB1), MRP1 (ABCC1), MRP2 (ABCC2), MRP3 (ABCC3), MRP4 (ABCC4), BCRP (ABCG2), PEPT2, OCT1, OCT3, OCTN2, OAT1, OAT2, OAT3, MATE1, OATP1A2, OATP1B1, OATP1B3, OATP2B1, NTCP and ASBT were measured using mass spectrometry-based targeted proteomics using validated LC−MS/MS. The membrane protein fraction was extracted from the tissue samples by the ProteoExtract^®^ Native Membrane Protein Extraction Kit (Merck KGaA, Darmstadt, Germany). Briefly approximately 30 mg of the crushed tissue samples were suspended in 500 µL cell lysing extraction buffer I (containing 5 µL/mL protease inhibitor cocktail) and incubated with shaking for 15 min at 4 °C. Subsequently, the homogenate was centrifuged at 16,000× *g* for 15 min at 4 °C. The supernatant (containing cytosolic proteins) was discarded, whereas pellets were resuspended in 300 µL extraction buffer II (containing 5 µL/mL protease inhibitor cocktail) and incubated with shaking for 60 min at 4 °C. In the next step, the suspension was centrifuged (15 min; 16,000× *g*; 4 °C), the supernatant was collected, and the whole protein concentrations in the membrane fractions were measured by bicinchoninic acid assay (Thermo Fisher Scientific, Schwerte, Germany). When needed, membrane fractions were adjusted to a maximum protein amount of 2 mg/mL. Afterwards, 100 µL of each membrane fraction was mixed with 10 µL dithiothreitol (200 mM, Sigma-Aldrich, Taufkirchen, Germany), 40 µL ammonium bicarbonate buffer (50 mM, pH 7.8, Sigma-Aldrich), and 10 µL ProteaseMAX™ (1%, *m*/*v*, Promega, Mannheim, Germany) and incubated for 20 min at 60 °C. After cooling, 10 µL iodoacetamide (400 mM, Sigma-Aldrich Taufkirchen, Germany) was added and the samples were maintained in a darkened water quench for 15 min at 37 °C (alkylation). Protein digestion was carried out by adding 10 µL of trypsin (trypsin/protein ratio: 1/40, Promega, Mannheim, Germany) followed by the sample incubation in a water quench for 16 h at 37 °C. Afterwards, 20 µL formic acid (10% *v*/*v*, Sigma-Aldrich, Taufkirchen, Germany) was added to stop the digestion. All samples were stored at −80 °C until processing. After thawing, 50 µL of the supernatant was mixed with 25 µL isotope-labeled internal standard (IS) peptide mix (10 nM of each IS; Thermo Fisher Scientific, Schwerte, Germany). Protein quantification was conducted on a 5500 QTRAP triple quadrupole mass spectrometer (AB Sciex, Darmstadt, Germany) coupled to an Agilent Technologies 1260 Infinity system (Agilent Technologies, Santa Clara, CA, USA) using validated LC–MS/MS methods as recently described [26]. Supplementary Table S4 summarizes the analyzed proteins and the respective protein specific peptides. 

During the analytical period, the accuracy of the method was within +/− 20% (relative error) as determined by analyzing quality control samples containing low (0.1 nmol/L), middle (1 nmol/L) and high (10 nmol/L) peptide concentrations, which were measured before, within and after the tissue samples. Final protein abundance data (picomoles per milligram) were calculated by normalization to the total protein content of the isolated membrane fraction as determined by the bicinchoninic acid assay (Pierce, Rockford, IL, USA). All samples were digested and measured in duplicate.

### 4.4. Immunofluorescent Analysis of MDR1, MRP1, OCT3, MATE1 and Na^+^/K^+^-ATPase 

Deparaffinized sections of salivary glands (3 μm thick) were hydrated, and heat epitope retrieval was performed in a microwave oven in retrieval solution buffer pH = 6 (DAKO, Denmark). After cooling to room temperature (RT), the slides were washed twice with PBS and further incubated with 2.5% horse serum (Vector Laboratories, Burlingame, CA, USA). After serum incubation, the slides were incubated with primary antibodies: rabbit polyclonal anti-human OCT3 (Sigma Aldrich, Taufkirchen, Germany), rabbit polyclonal anti-human MATE1 (Sigma Aldrich, Taufkirchen, Germany), mouse monoclonal anti-human MDR1 (Sigma Aldrich, Taufkirchen, Germany) and mouse monoclonal anti-human MRP1 (Abcam, Cambridge, UK) for 1 hour in RT, and after double washing in PBS, the slides were incubated with goat anti rabbit AlexaFluor 488 (Life Technologies, Waltham, MA, USA) for 1 hour at RT in the dark. After washing, the slides were incubated with mouse monoclonal anti-human Na^+^/K^+^-ATPase conjugated with AlexaFluor 647 (Santa Cruz Biotechnology, Dallas, TX, USA) for 1 hour at RT. After washing, nuclei were counterstained with DAPI (Sigma Aldrich, Taufkirchen, Germany) and mounted in fluorescent mounting medium (DAKO, Glostrup, Denmark). The same procedure was used to stain human kidney cortex slides, which served as positive control for OCT3, MATE1, MDR1 and MRP1 expression [50]. Images were collected with an Olympus IX81 inverted microscope (Olympus, Hamburg, Germany) and FV100 confocal system (Olympus, Hamburg, Germany). The slides were scanned separately for each channel, DAPI, AF488 and AF647, with a ×20 magnification objective and ×3.7 zoom during the scanning procedure. Images are shown as separate channels and merged, scale bar 30 μm.

### 4.5. Statistical Analysis

mRNA and protein expression data were presented as means (additional information: median, coefficient of variation, standard deviation, minimum and maximum value are given in Table 1 of the supporting information). Correlations between mRNA and protein level were assessed using the Spearman rank test. *p* values of <0.05 were considered significant. Statistical calculations were performed using STATISTICA 10 software package (StatSoft, Cracow, Poland).

## Figures and Tables

**Table 1:** Correlation between mRNA and protein level of ABC and SLC transporters in the human parotid salivary gland. Correlation coefficients were assessed using the Spearman’s rank test. Bold font indicates *p* value < 0.05.

mRNA vs. Protein	Salivary Gland
ABCC1	0.305
ABCC4	0.392
ABCG2	**−0.783**
PEPT2	0.000
OCT3	−0.133
MATE1	0.511
Na+/K+ATPase	0.017

## Supplementary Materials

Supplementary materials can be found at https://www.mdpi.com/1422-0067/20/19/4825/s1.
