# Supplementary material for: Membrane Transporters in Human Parotid Gland-Targeted Proteomics Approach
Source: Int J Mol Sci. 2019 Sep 28;20(19):4825. doi: 10.3390/ijms20194825 (PMC6801960; doi:10.3390/ijms20194825)
Supplement: Supplementary file 1 [file ijms-20-04825-s001.pdf]

Supplementary Table S1. Gene expression (mRNA relative quantity) of the ABC and SLC transporters in human parotid gland.

gene symbol	mean	SD	CV (%)	median	min	max
ABCB1	0.018	0.016	85.36	0.014	0.002	0.056
ABCC1	0.177	0.127	71.79	0.166	0.020	0.388
ABCC2	0.002	0.001	77.47	0.001	0.000	0.004
ABCC3	0.006	0.008	130.97	0.002	0.001	0.024
ABCC4	0.004	0.002	62.87	0.003	0.001	0.008
ABCG2	0.014	0.015	104.53	0.010	0.003	0.052
SLC10A1	BLQ					
SLC10A2	BLQ					
SLC15A2	0.063	0.064	101.58	0.049	0.009	0.224
SLC22A1	0.002	0.001	38.28	0.002	0.000	0.002
SLC22A3	0.029	0.032	109.22	0.023	0.001	0.106
SLC22A5	0.010	0.004	37.67	0.010	0.002	0.016
SLC22A6	BLQ					
SLC22A7	BLQ					
SLC22A8	BLQ					
SLC47A1	0.000	0.000	59.86	0.000	0.000	0.001
SLCO1A2	0.093	0.145	156.03	0.041	0.001	0.468
SLCO1B1	BLQ					
SLCO1B3	0.000	0.000	142.47	0.000	0.000	0.001
SLCO2B1	0.006	0.004	68.16	0.004	0.002	0.015
ATP1A1	1.575	0.499	31.68	1.534	0.781	2.660

Relative quantity of each transcript is presented, compared to mean expression of reference genes (ΔCT method); SD- standard deviation; CV % - coefficient of variation; BLQ-below limit of quantification.

Supplementary Table S2. Protein abundance of the ABC and SLC transporters in human parotid gland.

protein [pmol/mg]	mean	SD	CV (%)	median	min	max	positive samples
**ABCB1**	BLQ						
**ABCC1**	0.329	0.417	126.55	0.230	0.000	1.174	5/9
**ABCC2**	BLQ						
**ABCC3**	BLQ						
**ABCC4**	0.078	0.079	100.87	0.083	0.000	0.183	5/9
**ABCG2**	0.040	0.042	102.92	0.052	0.000	0.108	5/9
**NTCP**	BLQ						
**ASBT**	BLQ						
**PEPT2**	0.110	0.073	66.24	0.121	0.000	0.207	7/9
**OCT1**	BLQ						
**OCT3**	0.451	0.272	60.33	0.385	0.000	0.845	8/9
**OCTN2**	BLQ						
**OAT1**	BLQ						
**OAT2**	BLQ						
**OAT3**	BLQ						
**MATE1**	0.048	0.064	133.02	0.000	0.000	0.178	4/9
**OATP1A2**	BLQ						
**OATP1B1**	BLQ						
**OATP1B3**	BLQ						
**OATP2B1**	BLQ						
**Na/K ATPase**	7.908	7.030	88.89	8.400	0.575	18.727	9/9

SD- standard deviation; CV % - coefficient of variation; positive samples: number of samples with detectable level of the protein; BLQ-below limit of quantification.

Supplementary Table 3. Correlation between salivary glands transporters protein (Spearman's rank test). Bold fold indicates *p* value <0.05.

	ABCC1	ABCC4	ABCG2	PEPT2	OCT3	MATE1	OATP2B1	Na/K ATPase
**ABCC1**	1.000	**-0.773**	-0.009	-0.258	0.383	0.229	0.000	0.644
**ABCC4**		1.000	-0.127	-0.004	-0.052	-0.219	-0.358	**-0.957**
**ABCG2**			1.000	0.629	0.461	0.324	-0.358	0.174
**PEPT2**				1.000	0.042	0.440	0.138	-0.033
**OCT3**					1.000	0.183	-0.411	0.033
**MATE1**						1.000	0.300	0.164
**OATP2B1**							1.000	0.411
**Na/K ATPase**								1.000

Supplementary Table 4. Overview of used proteospecific peptides and the respective MS parameters.

Protein	Peptide	Q1	Q3.1	Q3.1-CE	Q3.2	Q3.2-CE	Q3.3	Q3.3-CE	DP	EP	CXP
**ABCB1**	AGAVAEEVLAAIR	635.4	971.4	27	430.2	23	900.3	27	80	10	13
	AGAVAEEVLAAIR*	639.5	981.4	27	440.2	23	910.3	27	80	10	13
	IATEAIENFR	582.4	749.4	30	565.2	28	678.4	28	70	10	13
	IATEAIENFR*	587.4	759.4	30	575.3	28	688.4	28	70	10	13
**ABCC1**	DGAFAEFLR	513.2	782.5	20	635.4	23	564.4	23	140	10	13
	DGAFAEFLR*	517.9	792.4	20	645.4	23	574.4	23	140	10	13
**ABCC2**	LTIIPQDPILFSGSLR	885.4	665.3	36	989.3	51	721.7	36	110	10	13
	LTIIPQDPILFSGSLR*	890.4	670.2	36	999.3	51	726.8	36	110	10	13
	YLGGDDLDTSAIR	698.5	547.3	43	1119.4	33	662.3	36	90	10	13
	YLGGDDLDTSAIR*	703.4	557.3	43	1129.4	33	672.3	36	90	10	13
**ABCC3**	IDGLNVADIGLHDLR	541.0	696.8	20	754.4	20	611.9	20	90	10	13
	IDGLNVADIGLHDLR*	544.3	701.8	20	759.3	20	616.8	20	90	10	13
	HIFDHVIGPEGVLAGK	563.9	749.5	27	650.4	29	513.4	37	151	10	13
	HIFDHVIGPEGVLAGK*	566.2	749.4	27	650.4	29	513.4	37	151	10	13
**ABCC4**	SSLISALFR	497.2	706.4	22	593.3	23	401.3	17	90	10	13
	SSLISALFR*	501.9	716.4	22	603.4	23	401.3	17	90	10	13
**ABCG2**	SSLLDVLAAR	522.8	644.3	23	757.3	23	430.3	22	80	10	13
	SSLLDVLAAR*	527.9	654.3	23	767.3	23	440.3	22	80	10	13
	VIQELGLDK	507.3	802.5	20	674.5	23	545.5	30	120	10	13
	VIQELGLDK*	511.3	810.6	20	682.5	23	553.5	30	120	10	13
**ASBT**	ENGTEPESSFYK	694.3	857.2	29	986.2	28	631.2	38	140	10	13
	ENGTEPESSFYK*	698.2	865.2	29	994.1	28	639.1	38	140	10	13
	AEIPESK	387.0	573.4	16	702.4	16	460.3	17	78	10	13
	AEIPESK*	391.1	581.4	16	710.4	16	468.2	17	78	10	13
	QEEPLPEHPQDGAK	525.7	594.9	21	615.4	31	650.6	24	140	10	13

**MATE1**	QEEPLPEHPQDGAK*	528.1	599.0	21	623.4	31	654.5	24	140	10	13
	GGPEATLEVR	514.94	457.9	23	617.3	23	688.4	30	65	10	13
	GGPEATLEVR*	519.9	462.8	23	627.3	23	698.4	30	65	10	13
**Na/K-ATPase**	LSLDELHR	328.3	435.2	15	391.7	14	669.3	15	100	10	13
	LSLDELHR*	331.5	440.3	15	396.8	14	679.4	15	100	10	13
**NTCP**	GIYDGDLK	440.7	710.4	18	547.2	19	432.2	25	60	10	13
	GIYDGDLK*	444.7	718.4	18	555.3	19	440.2	25	60	10	13
**OAT1**	TSLAVLGK	394.3	600.5	18	687.6	19	416.4	22	40	10	13
	TSLAVLGK*	398.3	608.5	18	695.6	19	424.3	22	40	10	13
**OAT2**	NVALLALPR	483.2	753.5	20	569.4	20	682.6	21	80	10	13
	NVALLALPR*	488.2	763.6	20	579.4	20	692.5	21	80	10	13
**OAT3**	VAVFNGK	367.3	564.3	15	635.3	17	465.4	18	60	10	13
	VAVFNGK*	371.2	572.4	15	643.5	17	473.3	18	60	10	13
**OATP1A2**	EGLETNADIIK	601.8	774.4	25	673.4	25	903.3	25	130	10	13
	EGLETNADIIK*	605.6	782.3	25	681.3	25	911.3	25	130	10	13
	YIYLGLPAALR	625.4	697.5	27	527.4	27	810.7	29	140	10	13
	YIYLGLPAALR*	630.3	707.6	27	537.4	27	820.5	29	140	10	13
**OATP1B1**	NVTGFFQSFK	587.9	961.4	24	860.4	24	656.4	27	70	10	13
	NVTGFFQSFK*	591.8	969.5	24	868.4	24	664.3	27	70	10	13
	SSIIHIER	477.8	554.3	26	667.4	26	417.2	30	80	10	13
	SSIIHIER*	482.8	564.3	26	677.4	26	427.2	30	80	10	13
**OATP1B3**	ISTIQIER	479.9	545.3	24	417.2	23	304.2	21	100	10	13
	ISTIQIER*	484.7	555.3	24	427.3	23	314.3	21	100	10	13
	NVTGFFQSLK	570.8	927.5	24	826.4	26	347.2	37	65	10	13
	NVTGFFQSLK*	574.9	935.5	24	834.5	26	355.2	37	65	10	13

**OATP2B1**	SSPAVEQQLLVSGPGK	798.8	711.9	33	445.2	35	1155.2	35	170	10	13
	SSPAVEQQLLVSGPGK*	803.7	716.7	33	453.1	35	1165.3	35	170	10	13
	YYNNDLLR	535.4	907.5	27	744.6	25	327.0	22	150	10	13
	YYNNDLLR*	540.2	917.6	27	754.5	25	327.0	22	150	10	13
**OCT1**	ENTIYLK	440.7	423.2	17	637.4	17	536.3	17	80	10	13
	ENTIYLK*	444.7	431.0	17	645.4	17	544.3	17	80	10	13
	LPPADLK	376.7	640.6	17	543.4	17	494.3	14	50	10	13
	LPPADLK*	380.9	648.4	17	551.4	17	494.3	14	50	10	13
**OCT3**	GIALPETVDDVEK	693.3	1031.3	25	516.2	28	805.2	38	120	10	13
	GIALPETVDDVEK*	697.3	1039.3	25	520.3	28	813.3	38	120	10	13
**OCTN2**	FEEAEVIIR	552.9	829.4	26	700.5	27	629.4	26	160	10	13
	FEEAEVIIR*	558.1	839.6	26	710.5	27	639.5	26	160	10	13
**PEPT2**	IEDIPANK	450.3	657.4	19	429.2	27	786.5	20	110	10	13
	IEDIPANK*	454.1	665.4	19	437.3	27	794.6	20	110	10	13

Peptides highlighted in grey were used for quantification (CE, collision energy; DP, declustering potential; EP, entrance potential; CXP, collision cell exit potential; Q, quadrupole).
